# Identifying and Overcoming Barriers to Resident Use of Point-of-Care Ultrasound

**DOI:** 10.5811/westjem.2019.8.43967

**Published:** 2019-10-14

**Authors:** Nikolai Schnittke, Sara Damewood

**Affiliations:** * Oregon Health and Science University, Department of Emergency Medicine, Portland, Oregon; † University of Wisconsin School of Medicine and Public Health, Department of Emergency Medicine, Madison, Wisconsin

## Abstract

**Introduction:**

Emergency medicine residency programs have rigorous point-of-care ultrasound (POCUS) curricula. However, this training does not always readily translate to routine use in clinical decision-making. This study sought to identify and overcome barriers that could prevent resident physicians from performing POCUS during clinical shifts.

**Methods:**

This was a two-step process improvement study. First, a survey was deployed to all residents of a three-year academic residency program to identify barriers to clinical use of POCUS. This survey identified the perceived lack of a uniform documenting protocol as the most important barrier to performing POCUS on shift. Second, as an intervention to overcome this barrier, a streamlined documentation protocol was developed and presented to residents. The primary outcome was the number of patients who had POCUS used in medical decision-making one year before and after intervention. Secondary outcomes were the level of training of residents performing exams and whether faculty overseeing exams were trained through an ultrasound fellowship program.

**Results:**

POCUS use by residents increased from 82 to 223 patients before and after the intervention, respectively. Per resident, this translates to an absolute increase from 2.2 (95% confidence intervall [CI], 1.4, 3) to 5.8 (95% CI, 4, 7.6) or 3.6 (95% CI, 1.8, 5.4) exams/resident over the study period. We observed no significant difference in the proportions of scans attributable to the resident level of training (χ2 = 0.5, p = 0.47). The proportion of exams by non-ultrasound fellowship trained faculty increased significantly more compared to fellowship trained faculty (χ2 = 19, p<0.0001); however, both ultrasound fellowship trained and non-ultrasound fellowship trained faculty increased the absolute number of exams performed.

**Conclusion:**

A key perceived barrier to resident-performed POCUS is unfamiliarity with documenting ultrasounds for medical decision-making. Educating residents in person about a POCUS documentation protocol may help overcome this barrier. Incorporating resident input and motivation into POCUS incentivization may increase utilization. Future studies in optimizing POCUS on shift will need to focus on streamlining documentation, addressing time constraints, and faculty support for resident-performed POCUS.

## INTRODUCTION

Point-of-care ultrasound (POCUS) has emerged as an essential diagnostic tool in emergency medicine (EM). 1 Several studies have demonstrated that a structured curriculum is both feasible and effective in training emergency physicians (EP) to obtain and accurately interpret images with test characteristics approaching or even exceeding those of dedicated radiology-performed scans. 2–5 However, less is known about the penetrance of POCUS into daily EP practice. The emergency department (ED) poses unique challenges to implementation of diagnostic POCUS not present in other specialties with broad adoption of POCUS such as cardiology, critical care, and obstetrics: 1) the time spent with an individual patient is limited compared to other specialties; 2) ED settings vary dramatically between academic, community, rural, and urban practices, and each environment has its own unique challenges with respect to availability of POCUS and training of clinicians in ultrasound; 6 and 3) the breadth of POCUS applications in the ED is considerably greater than in other specialties.

Guidelines from the American College of Emergency Physicians (ACEP) endorse 12 core applications. The degree of experience necessary to obtain competency in image acquisition and interpretation, while not clear, appears to be highly variable between these applications. 7,8 As a result, few EPs maintain competency in all 12 applications without further postgraduate fellowship training. This leads to a general reluctance to perform and rely on some POCUS exams, as EPs question the need to maintain competency in certain applications. 9 Indeed, a survey of EPs in California found that most EPs do not use POCUS, and that EPs in academic environments use POCUS more regularly than their community counterparts. 10

The challenges posed above apply both to established EPs and residents in training who are establishing practice patterns. Despite near-universal incorporation of ultrasound into resident training, 11 a survey of recent residency graduates found limited use in daily clinical practice. 12 This suggests that dedicated ultrasound training in most EM residency programs in North America progresses residents to the intermediate level, where they are able to effectively acquire and interpret images, but not to the level of the expert who is able to seamlessly incorporate the procedural skill into practice. We hypothesized that a number of perceived barriers may be leading to a gap in deliberate, on-shift practice, which is preventing trainees from advancing to expert levels.

The goal of this study was to assess and address relevant barriers to POCUS performed on shift by residents at a single, three-year EM residency program. As such, the study had two phases. We first performed a voluntary residency-wide survey to address perceived attitudes and barriers to on-shift use of POCUS. Next we performed an intervention to address the primary barrier, namely the perceived lack of a proper charting and reporting policy.

## METHODS Setting

We conducted the study at an ED with an annual volume of 65,000 patients, which hosts a three-year EM residency program. The residency trains a total of 36 residents, with 12 residents per year. The study site uses the HealthLink/EPIC electronic medical record (Epic Systems, Verona, WI), and all point-of-care ultrasounds are wirelessly uploaded to a middleware product (Q-Path, Telexy Health Systems, Seattle, WA). Quality assurance of all scans submitted for review is performed by ultrasound fellowship-trained EPs who rotate on a weekly basis.

**Population Health Research Capsule**

What do we already know about this issue?

*Ultrasound is an essential component of emergency medicine resident education, yet its use in emergency physicians’ daily practice remains relatively low*.

What was the research question?

What are the barriers to resident use of ultrasound in clinical practice?

What was the major finding of the study?

*After an educational intervention on ultrasound documentation, ultrasound utilization increased*.

How does this improve population health?

*Addressing barriers to clinical ultrasound use allows emergency physicians to fully use the benefit of this imaging modality in the care of emergency patients*.

At the time of study performance, ultrasound training consisted of a four-hour introductory ultrasound course at the start of residency training, a four-week mandatory ultrasound rotation during the first year, and quarterly didactics with simulation and hands-on training during regularly scheduled mandatory conference. In addition, ultrasound fellowship-trained faculty offered three-hour sessions, biweekly, which consisted of didactics, image review, and bedside scanning. These sessions were mandatory for the first-year resident who was on the dedicated POCUS rotation, as well as two second- and third-year residents who were on a dedicated month of community ED practice. The study was performed as part of ongoing quality improvement (QI) program, not requiring institutional review board review at the study institution.

### Workflow

At the beginning of the study, a departmental best-practice, systematic, ultrasound documentation workflow was disseminated to faculty attending physicians. This workflow included saving ultrasound examinations performed or supervised by a faculty member credentialed in the relevant application. The images were then transferred from Q-Path to the hospital picture archiving and communication system (PACS) where they are visible to all hospital providers. Finally, the findings were documented under the “Procedures” section of the ED provider note, and referenced in the medical decision-making portion of the note as appropriate. Pre-established macros (smart-phrases) for documentation of each application were shared with all providers. All faculty were credentialed in accordance with ACEP guidelines. Under this policy, residents may perform the ultrasound exam under supervision of credentialed faculty and submit scans to count toward their own credentialing.

### Survey

The survey was designed with input from interviews with faculty, including residency and QI leadership, and residents. The survey was sent out using the online SurveyMonkey tool. Paper print-outs of the survey were also made available to residents to facilitate compliance. The resident performing the study (NS) was excluded from this survey. Responses were weighted using a five-point Likert scale.

### Intervention

Following collection of survey responses, a 15-minute presentation was given to the residents on January 7, 2016, outlining the new charting policy, by the resident organizer of the study (NS). The presentation outlined the workflow for using ultrasound in clinical management, including appropriate charting procedure. The results of the survey were also shared with faculty via email. These interventions were timed to coincide roughly with the middle of the academic year which spans the time period of July 2015–July 2016.

### Data Collection

We collected data for all ultrasound exams used for clinical decision-making for one year pre- (January 7, 2015–January 7, 2016) and post-intervention (January 8, 2016–January 8, 2017). Only the residents who were part of the residency program at the time of the intervention were included in the study. Prior to data collection we set the primary outcome as the number of scans submitted to PACS. As secondary outcomes we analyzed the involvement of general compared to POCUS fellowship-trained faculty, and the level of training of the residents performing the scans.

## RESULTS

### Survey

**Figure 1:**
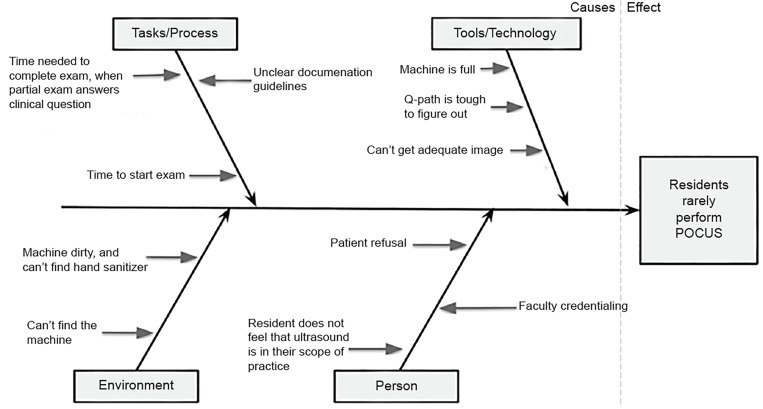
“Fish-bone” diagram derived from qualitative assessment of potential barriers to clinical use of point-of-care ultrasound (POCUS) by emergency medicine residents.

At the beginning of the study, we performed a qualitative needs assessment with a workgroup, including the authors of the study, residency leadership, QI leadership, and unstructured interviews with residents. We generated potential contributors to the observation that residents rarely use POCUS on shift and summarized them in a “fish-bone” diagram (Figure 1). Based on this list, we created a survey of residents to help further elucidate residents’ attitude towards POCUS and the leading barriers to POCUS use on shift (Supplemental Appendix).

Participation in the survey was voluntary, and we received responses from 27/35 (77%) residents with comparable contribution from residents at all three levels of training. We found that 30% of all residents reported never using POCUS on shift, 52% reported using POCUS approximately once per shift, and 18% used POCUS more than once per shift. When asked about general attitudes toward ultrasound use and training, most residents somewhat agreed or strongly agreed that ultrasound is an important skill for residents to learn (96%) and practice in our ED (93%). Most residents also somewhat agreed or strongly agreed that POCUS will be important in their future practice (92%). However, responses were somewhat tempered in considering whether availability of POCUS would be important in their search for future employment: 63% somewhat or strongly agreed, while 7% somewhat disagreed (Table 1).

In assessing barriers to on-shift use of ultrasound we found that the “inability to use results in documentation” received the highest weighted average rating of 3.7 on a five-point Likert scale with 41% and 25% of residents, respectively, reporting that this was a significant and extreme barrier. Time barriers, including time to complete/optimize exams and time required to initiate an exam were also rated highly with weighted averages of 3.6 and 3.2. Barriers pertaining to tools and technology such as Q-Path navigation, inability to find the machine, space on the machine, and gel availability were generally ranked as only “slight barriers” with weighted average scores of 2.2, 2.1, 1.8, and 1.6, respectively (Table 2).

Finally, we attempted to assess potential incentives that would help residents overcome the barriers above. We found that increased attending support was the top perceived incentivizer for residents with a weighted average of 4. Residents also felt that clear guidelines on charting were likely to incentivize scanning (weighted average score of 3.8).

### Effects of Intervention

**Figure 2:**
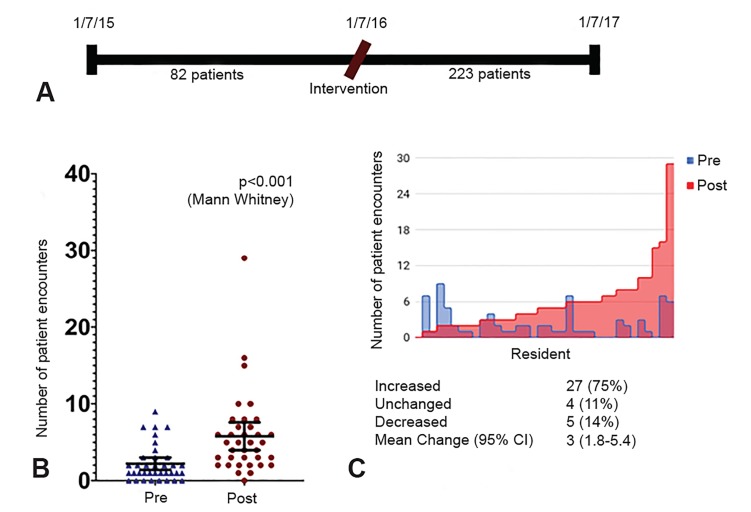
Implementation and education of an ultrasound documentation policy increases point-of-care ultrasound utilization by residents. A. Timeline of the study period and observed increase in billed scans from 82 to 223. B. Mean patients scanned per resident increased by an average 3.6 patients/resident. Error bars = 95% confidence intervals. Each data point represents individual residents C. Evaluation of number of patients scanned pre- and post-intervention by each individual resident (where each resident is represented by a vertical bar). The majority (75%) of residents increased their ultrasound use after intervention. *CI*, confidence interval.

Following completion of the survey, we designed an intervention aimed at addressing the highest-scoring barrier to on-shift POCUS, namely the perceived lack of the ability to document scans in medical-decision making. This intervention involved an in-person education session during resident conference on an established guideline for on-shift documentation. The guideline was also disseminated to residents and faculty via email. We found that significantly more patients received at least one POCUS exam performed on shift and used in medical decision-making in the year following the intervention (223) compared to prior to the intervention (82) (Figure 2A). Per resident, this corresponds to an absolute increase from 2.2 (95% confidence interval [CI], 1.4, 3) to 5.8 (95% CI, 4, 7.6) or 3.6 (95% CI, 1.8, 5.4) exams/resident over the study period (p<0.0001, Mann-Whitney U-test) (Figure 2B). We also looked at the number of patients scanned by each resident and found that the majority of residents (75%) increased their scanning, while only 14% of residents decreased their scanning, suggesting that the effect size was not due solely to outlier residents (Figure 2C).

**Figure 3:**
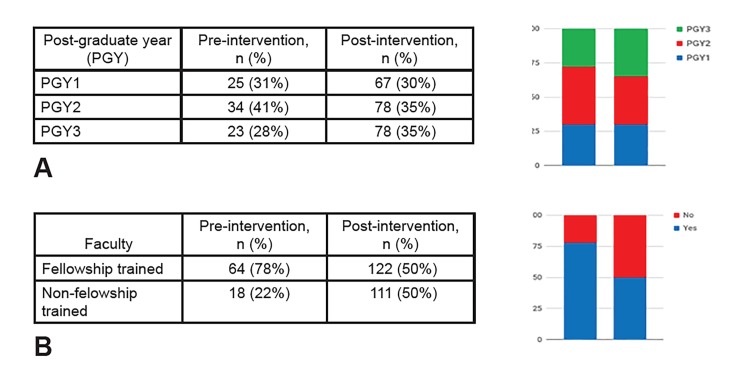
Secondary outcome analysis. A. Subgroup analysis of individual residency classes by postgraduate (PGY) year, showing no significant difference between PGY level and increase in point-of-care ultrasound utilization. B. Subgroup analysis of faculty. The non-ultrasound trained faculty demonstrated a significant increase in the total proportion of exams performed compared to the ultrasound trained faculty.

In assessing secondary outcomes, we found no significant difference in the proportion of scans performed by residents at various postgraduate year levels (χ2=0.5, p=0.47) (Figure 3A). In addition, while POCUS fellowship-trained faculty performed more scans than non-fellowship trained faculty both pre- and post-intervention, the total proportion of scans performed increased significantly more in the non-fellowship trained faculty cohort from 22% to 50% (χ2=19, p<0.0001) (Figure 3B).

## DISCUSSION

Ultrasound training is a core feature of EM residency training. However, there is a considerable variability in the form this training takes throughout residencies in the United States. 13 In order to characterize POCUS training of EM residents, Hayward et al. applied Ericsson’s deliberate practice model of acquiring procedural proficiency. This model divides learners into novice, intermediate, expert, and advanced expert levels who are able to learn the basics, apply them efficiently, apply them intuitively, and apply advanced applications of the procedure respectively. 14

To advance trainees from intermediate sonographers (ie, ones who are competent in image acquisition and interpretation) to expert sonographers (ie, ones who seamlessly integrate POCUS into daily practice and patient care), one must have a detailed understanding of the barriers to such a transition. To the best of our knowledge, this study is the first attempt to systematically define and address these barriers in a resident population. Our data highlight a number of key findings, likely relevant to curriculum and POCUS workflow design:

First, we found that residents’ perception of ultrasound and its importance in modern EM training is overwhelmingly positive with 96% of residents believing that ultrasound is an important skill to learn during their training. Despite this, only 63% of residents believed that ultrasound availability would be an important feature for them in their future job search. This discrepancy likely underscores the larger problem posed above: While residents are enthusiastic and competent in image acquisition and interpretation, next level training in methods of integrating ultrasound into daily practice is lacking.

Second, we were somewhat surprised that the major barrier identified by residents at the time of our study was the perceived inability to use ultrasound for medical decision-making rather than conventional barriers of time available in the ED or equipment malfunction. However, when viewed through the lens of the deliberate practice model of transitioning from intermediate to advanced competency, it makes sense that our residents’ grasp on how to use ultrasound in daily practice was the major perceived barrier.

Third, our finding that implementation and education of a documentation policy is associated with increased integration of ultrasound in clinical decision-making has significant implications for resident education and its integration into subsequent ED ultrasound billing workflows. Recent studies have demonstrated that a continuous workflow quality improvement efforts for all staff also significantly increased the proportion of reported and billed ultrasound studies. 15,16 Another recent study found that resident education of billing practices significantly increased RVU billable by resident encounters. 17 Taken together, this body of literature suggests that educational interventions such as ours can have a quantifiable effect on ED revenue and future EP documentation practices.

A potential confounder in the before-after design of our study was a concomitant push for faculty credentialing, which was underway in our department during the study period. To assess whether the increase in the patients scanned may have been due to this confounder we also analyzed the number of POCUS studies uploaded to PACS by faculty ***without*** resident involvement. We found that faculty uploaded 124 vs 138 studies, which were done without resident involvement, during the pre- and post-intervention phases of the study, an absolute increase of 6%, while resident scans uploaded to PACS increased by 78% (p<0.0001, Fischer’s exact test). Thus, it appears that the increase in scans performed was primarily resident-driven.

Finally, while it is difficult to infer causation in this observational, before-after study, it does provide a suggestion that incentivization of residents and faculty might be linked. Our secondary outcome demonstrated that the resident-based intervention increased scanning among non-fellowship trained faculty, more so than among ultrasound fellowship-trained faculty. As methods of faculty credentialing and education continue to advance, it may be useful to integrate resident and faculty education. Future inquiry into the effect and interplay of faculty and resident incentivization may help make the transition from intermediate to advanced sonographer more robust and efficient.

## LIMITATIONS

This study was performed at a single academic center with an EM residency program, and as such may be limited in external applicability. However as mentioned earlier, our institution faces many of the same problems and barriers that have been reported by other institutions in the literature. These include the low rate of POCUS utilization, need for deliberate practice, implementation of intuitive documentation processes, and lack of time in a busy ED. 15

While we did solicit feedback from residency leadership and residents, within the limitations of a single-center quality improvement study, we did not perform separate validation of the survey. The survey portion was also subject to sampling bias, since we had only a 77% response rate. However, we believe that voluntary and anonymous reporting on the survey provides a sufficient advantage. Our low sample size, given its single-center nature, is an important limitation as it limits the statistical power of the study, and it would be useful to repeat this study on a nationwide level. The survey itself includes closed-ended questions, which may introduce response bias; however, write-in, free-text responses were allowed.

In regard to our primary outcome, our study may be limited by the assumption that the number of exams uploaded to PACS is an accurate marker for the number of scans used in the medical decision-making process. Indeed, the survey responses suggest that 82% of residents used POCUS one or more times per shift, but even after the intervention there were only 5.8 scans documented per resident. This suggests that a large proportion of POCUS studies are never documented (a phenomenon often referred to as “scan and run” or “phantom scans”). In addition, this surrogate marker also relies on the cooperation of the appropriate attending, as residents did not have ability to upload images to PACS. However, the survey does identify lack of documentation ability as an important barrier, and documentation of POCUS studies is essential to appropriate medical decision-making and billing as laid out in ACEP’s clinical guideline on POCUS use. Thus, our study’s primary outcome is relevant to the key objective of the study (ie, facilitating POCUS use in clinical practice).

Another key limitation of our study is the before-after design, which introduces a number of confounders. During the study period faculty received ongoing reminders and were actively incentivized to increase clinical use of POCUS. It is unlikely that the increase in scans is due solely to our intervention; however, we found that the increase in resident-performed POCUS studies is disproportionate to the number of studies done by faculty alone, suggesting that resident involvement in POCUS documentation should be a key factor in improving the quality of POCUS use in clinical decision-making.

## CONCLUSION

This work demonstrates that residents in our program perceive POCUS as valuable to their practice of EM, but recognize a number of barriers to routine incorporation into clinical care. Unfamiliarity with documentation procedure was a key barrier to resident use of POCUS on shift, and addressing this barrier with in-person education helped improve the number of ultrasounds used in medical decision-making. Future work is warranted to establish user-friendly documentation procedures and evaluate the mechanisms of knowledge translation necessary to transition competent resident level sonographers into advanced attending level sonographers.

## Supplementary Information



## Figures and Tables

**Table 1:** Resident attitudes toward point-of-care ultrasound (POCUS) education and use.

How do you feel about POCUS?	Strongly disagree	Somewhat disagree	Neutral	Somewhat agree	Strongly agree	Weighted average
Ultrasound is an important skill for residents to learn	0	0	1	5	21	4.74
Ultrasound is an important skill to practice in our emergency department	0	0	1	12	14	4.48
Ultrasound will be an important part of my future emergency medicine practice	0	0	2	12	13	4.41
Ultrasound availability will be important for me when I look for a job	0	2	8	11	6	3.78

**Table 2:** Perceived barriers to routine use of ultrasound in clinical practice.

Potential Barriers	Not a Barrier	Slight Barrier	Moderate Barrier	Significant Barrier	Extreme Barrier	Weighted average
Inability to use the results in documentation	0	6	3	11	7	3.7
Time to complete/optimize a full exam	0	5	5	12	5	3.63
Available time to start an exam	3	4	8	7	5	3.26
Radiology ultrasound too readily available	4	4	9	6	4	3.07
Not knowing if your attending is credentialed	4	4	14	4	1	2.78
Difficult to figure out Q-Path	8	9	6	4	0	2.22
Can’t find the ultrasound machine	7	13	5	1	1	2.11
The machine is out of space	12	9	5	1	0	1.81
Can’t find gel	13	11	2	0	0	1.58
Patient refusal	18	5	3	1	0	1.52
You don’t see it as within your scope of practice	22	4	1	0	0	1.22
