# Supplementary material for: Identifying and Overcoming Barriers to Resident Use of Point-of-Care Ultrasound
Source: West J Emerg Med. 2019 Oct 14;20(6):918–25. doi: 10.5811/westjem.2019.8.43967 (PMC6860390; doi:10.5811/westjem.2019.8.43967)
Supplement: Supplementary file 1 [file wjem-20-918-s001.docx]

POCUS

1. What is your level of training?
2. 1st year, completed US rotation
3. 1st year, not yet completed US rotation
4. 2nd Year
5. 3rd Year
6. On average, how many times per shift do you put an US probe on a patient?
7. Never
8. Once
9. More than once
10. I slap that baby on every time someone comes in with SOB, leg swelling, headache, right upper quadrant pain, vaginal bleeding, abdominal pain, flank pain, low blood pressure, sepsis, or trauma
11. How do you feel about point-of-care ultrasound

|  | Strongly Disagree | Somewhat Disagree | Neutral | Somewhat Agree | Strongly Agree |
| --- | --- | --- | --- | --- | --- |
| POCUS is an important skill for residents to learn |  |  |  |  |  |
| POCUS is an important skill to practice in our ED |  |  |  |  |  |
| POCUS will be an important part of my future EM practice |  |  |  |  |  |
| POCUS availability will be important for me when I look for a job |  |  |  |  |  |

1. Which of the following indications do you anticipate using as an a attending? (cicrle all that apply)

Cardiac (including IVC)

Thoracic/lung

Aortic

Renal

DVT

Hepatobiliary

Soft tissue/MSK

Ocular

Pregnancy

Procedural guidance (including central lines)

Trauma

1. Please rate the following barriers to performing POCUS

|  | Not a Barrier | Slight Barrier | Moderate Barrier | Significant Barrier | Extreme Barrier |
| --- | --- | --- | --- | --- | --- |
| Available time to start an exam |  |  |  |  |  |
| Time to complete/optimize a full exam |  |  |  |  |  |
| Inability to use in the results in documentation |  |  |  |  |  |
| Not knowing if your attending is credentialed |  |  |  |  |  |
| Difficult to figure out Q Path |  |  |  |  |  |
| Can’t find the US machine |  |  |  |  |  |
| Can’t find the gel |  |  |  |  |  |
| The machine is out of space |  |  |  |  |  |
| Radiology US too readily available |  |  |  |  |  |
| Patient refusal |  |  |  |  |  |
| You don’t see it as within your scope of practice |  |  |  |  |  |

1. How likely are the following interventions to get you to do POCUS on shift

|  | Not Likely | Somewhat unlikely | Neutral | Somewhat Likely | Very Likely |
| --- | --- | --- | --- | --- | --- |
| Some kind of incentive for SUBMITTED complete scans regardless of QA APPROVAL (independent of credentialing/graduation requirements) |  |  |  |  |  |
| Attendings supporting POCUS on shift for MDM |  |  |  |  |  |
| Displaying attending US certifications in EPIC |  |  |  |  |  |
| Displaying attending US certifications on the US machine |  |  |  |  |  |
| Clear guidelines on charting |  |  |  |  |  |
| Conference attendance credits |  |  |  |  |  |
| Class dinner for class with most submitted scans |  |  |  |  |  |
| Location trackers on machines |  |  |  |  |  |

1. Are you familiar with the .EDUS_ procedure dot-phrase? Yes / No
2. Are there any other barriers to you doing POCUS?
3. Do you have any other suggestions to overcome barriers to POCUS?
